# Adolescents’ Psychological Inflexibility as a Mediator Between Maternal Inflexibility and Internalizing Problems

**DOI:** 10.3390/children12081000

**Published:** 2025-07-30

**Authors:** Luisa Fanciullacci, Paolo Pricoco, Marco A. Malanima, Marco Fornili, Laura Baglietto, Martina Smorti, Carmen Berrocal

**Affiliations:** 1 Department of Clinical and Experimental Medicine, University of Pisa, 56126 Pisa, Italy; l.fanciullacci@studenti.unipi.it (L.F.); p.pricoco@studenti.unipi.it (P.P.); marco.malanima@phd.unipi.it (M.A.M.); marco.fornili@unipi.it (M.F.); laura.baglietto@unipi.it (L.B.); 2 Department of Surgical, Medical and Molecular Pathology and Critical Care Medicine, University of Pisa, 56126 Pisa, Italy; martina.smorti@unipi.it

**Keywords:** psychological inflexibility, ACT, internalizing disorders, adolescence

## Abstract

**Background/Objectives**: Internalizing disorders are highly prevalent during adolescence. Previous research has shown that psychological inflexibility (PI) in both adolescents and their parents contributes to internalizing problems. However, how parental and adolescent PI relate to one another in explaining these difficulties remains unclear. The present exploratory study examined whether adolescent PI mediates the relationship between maternal PI and internalizing problems in adolescents. **Methods**: The study sample included 81 mother–adolescent dyads (80% female adolescents). Mothers completed a general self-report measure of PI, while adolescents completed self-report measures assessing both PI and internalizing problems. **Results**: The results showed that adolescent PI partially mediated the relationship between maternal PI and internalizing difficulties in female adolescents, whereas the mediation model was not significant for male adolescents. **Conclusions**: Findings suggest that mothers with high levels of psychological inflexibility may foster similar patterns in their children, particularly in daughters, thereby increasing adolescents’ vulnerability to increased psychological distress.

## 1. Introduction

Internalizing problems refer to the presence of depressive, anxious, and somatic symptoms [1,2]. These difficulties are highly prevalent during adolescence [3,4,5,6,7], particularly among youth aged 15–19 [8]. Gender is also a significant risk factor, with female adolescents being 2.9 times more likely to experience internalizing problems than their male peers, e.g., [4,5,7,9,10,11,12,13].

The psychological inflexibility (PI) model, rooted in third-wave cognitive-behavioral approaches, has become an important framework for understanding the development and maintenance of a wide range of psychological difficulties, including internalizing disorders [14,15,16,17,18]. The PI model identifies PI as a key transdiagnostic process contributing to mental health problems. PI refers to the rigid dominance of internal experiences (e.g., thoughts, emotions, and memories) over personal values and direct contingences in guiding behavior [14]. Several interrelated processes have been proposed to underlie PI, including experiential avoidance (i.e., rigid attempts to avoid internal experiences such as unpleasant thoughts and emotions), cognitive fusion (i.e., rigid regulation of behavior through verbal processes), attachment to a conceptualized self, attentional rigidity, lack of clarity about personal values, and generalized patterns of behavior that are inconsistent with one’s values [14].

Acceptance and Commitment Therapy (ACT) [19] is a psychological intervention designed to reduce PI and foster psychological flexibility (PF). PF refers to the capacity to remain in contact with the present moment and to change or maintain behavior in ways that align with personally meaningful values, depending on situational demands [14,19]. PF involves six interrelated processes that support adaptive functioning: acceptance of unpleasant internal experiences, cognitive defusion, present-focused and flexible attention, a sense of self-as-context, clarity about personal values, and committed action [14,19]. Together, these processes help individuals respond to difficult internal experiences with openness and awareness, reduce entanglement with distressing thoughts and emotions, and promote consistent action in line with personal goals and values.

A growing body of research indicates that higher levels of PI are positively associated with increased internalizing problems in children and adolescents aged 8–20 years from the general population [20,21,22,23,24,25,26,27,28]. Furthermore, longitudinal studies have shown that specific components of PI, such as experiential avoidance and cognitive fusion, predict depressive and anxiety symptoms over time in youth [29,30]. Specifically, Valdivia-Salas et al. [29] found that higher levels of cognitive fusion assessed at baseline significantly predicted increased depressive symptoms four weeks later in secondary school students (51% females) aged between 11 and 17 years. Similarly, Mellick et al. [30] demonstrated that higher levels of experiential avoidance predicted both depressive and anxiety symptoms an average of 342.1 days later in adolescents (61.7% female) aged 15 to 20 years. Furthermore, a recent review by [31] suggests that, although still preliminary, ACT appears to be a promising intervention for reducing anxiety and depression in adolescents aged 12 to 17 years.

In addition to individual PI, several studies have found that parents’ PI is also significantly associated with internalizing difficulties in their offspring [32,33]. Specifically, Brassell and colleagues [32] found that higher levels of parental PI were associated with greater internalizing problems in their offspring in a sample of parents of adolescents aged 13 to 17 years (37.1% female). Similarly, Daks et al. [33] reported that greater parental PI was correlated with higher levels of child distress in a sample of parents of adolescents aged 5–18 years (50% female). It is important to note that in both studies, internalizing difficulties were assessed exclusively through parent-report measures, which may have introduced bias related to parental perception.

Taken together, these findings suggest that PI may operate not only at the individual level, but also within family dynamics, potentially contributing to the intergenerational transmission of emotional distress. However, the relationship between parents’ and adolescents’ PI in explaining internalizing problems remains unclear. Except for the study by Moyer and Sandoz [34], which will be discussed below, previous research has examined psychological outcomes in relation to either parental or adolescent PI separately, but rarely both concurrently. Theoretically, it is plausible to hypothesize that adolescent’s PI mediates the association between parents’ PI and adolescent’s internalizing problems. This is consistent with the idea that psychologically inflexible parents may engage in less effective parenting strategies that hinder the development of psychological flexibility in their children. For example, parental inflexibility has been associated with emotionally avoidant communication styles [35], the use of coercive emotional control (e.g., inducing guilt or fear) [36], and inconsistent or rigid discipline strategies [32], all of which may foster PI in youth.

Although not testing mediation directly, Moyer and Sandoz [34] examined the association between parents’ and adolescent’s PI in a sample of 21 parent–offspring dyads and found no significant relationship. The authors suggested that this finding might be due to their small sample size and the use of a specific questionnaire assessing parents’ PI exclusively within the context of parenting. Furthermore, adolescent gender was not considered in their analyses. Given the higher prevalence of both internalizing problems (e.g., [4,7,9]) and PI [37,38] among female adolescents, stratifying analyses by gender may help clarify potential gender-specific pathways linking PI and psychopathology.

The present study aimed to further examine the relationship between maternal and adolescents’ PI, and to test whether adolescent PI mediates the association between maternal PI and adolescent’s internalizing problems. To our knowledge, this is the first study to empirically test this mediational hypothesis. While exploratory and preliminary, the study advances previous related research [34] by employing a generic (rather than a parenting-specific) measure of PI in adults, using a larger sample of parent–offspring dyads, and considering the role of adolescent gender in these relationships.

## 2. Materials and Methods

### 2.1. Participants and Procedure

This study adopted an observational cross-sectional design. Ethical approval was obtained by the Bioethics Committee of the University of Pisa, Italy. Participants were mother–adolescent dyads recruited in 2023 from three secondary schools and first-year psychology university courses in the Tuscany region (Italy). The study was presented directly to students in the classroom setting. Students also received an information sheet inviting both themselves and their parents to participate. Participation involved completing a battery of online self-report questionnaires, accessible through a QR code, and described below. All participants were required to sign an informed consent form. In the case of minors, consent was also obtained from their parents. Participation was anonymous and voluntary, and no compensation was offered.

### 2.2. Measures

The research protocol comprised an initial section to collect socio-demographic data (i.e., gender, age, education level, nationality, and mother’s employment and marital status), followed by a section assessing the study variables through the self-report questionnaires described below.

The adolescents completed the Avoidance and Fusion Questionnaire for Youth (AFQ-Y [39]; Italian version by [40]) and the Youth Self Report (YSR [41]; Italian version by [42]).

The AFQ-Y is a self-report questionnaire designed to measure PI in children and adolescents. It consists of 8 items rated on a 5-point Likert scale ranging from 0 (“not at all true”) to 4 (“absolutely true”), with higher scores reflecting greater levels of PI. The AFQ-Y has demonstrated adequate internal consistency and good criterion validity, as shown by its concurrent associations with anxiety, depression, and somatic symptoms [39,40]. In the present study, Cronbach’s alpha coefficient was 0.77.

The YSR is a widely used self-report questionnaire developed to assess internalizing and externalizing problems during childhood and adolescence. It includes 112 items rated on a 3-point Likert scale ranging from 0 (“not true”) to 2 (“very true or often true”). The YSR yields five subscales grouped into two broadband dimensions: internalizing (comprising withdrawal, anxiety/depression, and somatic complaints) and externalizing (comprising aggressive and rule-breaking behavior). The internalizing subscale was used in this study. The YSR, widely validated in both clinical and non-clinical populations, has demonstrated good internal consistency as well as solid concurrent and discriminant validity in the assessment of internalizing problems [43,44]. Cronbach’s alpha coefficient was 0.91 in this sample.

The mothers completed the Acceptance and Action Questionnaire-II (AAQ-II [45]; Italian version by [46]). The AAQ-II is a self-report questionnaire developed to measure PI in adults. It consists of 7 items rated on a 7-point Likert scale ranging from 1 (“never true”) to 7 (“always true”), with higher scores indicating greater PI. The AAQ-II has shown high internal consistency and good concurrent and convergent validity with measures of anxiety, depression, and psychological well-being in general population samples [46]. In the current study, Cronbach’s alpha coefficient was 0.89.

### 2.3. Statistical Analysis

Categorical variables were summarized by counts and percentages, and continuous variables by means and standard deviations. Fisher’s exact tests and Welch’s *t*-tests for unequal variances were performed to assess group differences by gender. All the statistical analyses were stratified by the adolescent’s gender. Spearman correlation coefficients were calculated to examine associations among continuous variables. In all regression and mediation analyses, continuous variables were standardized. Multiple linear regression analyses were conducted to test the predictive role of mother’s and adolescent’s PI on internalizing problems. Finally, mediation analyses were performed to examine whether adolescent PI mediated the effect of mothers’ PI on internalizing problems. Mediation analyses were conducted with maternal PI treated both as a continuous and a dichotomous variable (lower versus higher than the mean), in order to account for potential deviations from linearity. The Akaike Information Criterion (AIC) was used to compare the models including maternal PI as continuous or dichotomous. All regression and mediation analyses were adjusted for the mother’s and adolescent’s age. All statistical tests were two-sided. All statistical analyses were performed with R, version 4.4.3.

## 3. Results

The sample included 81 mother–offspring dyads. Sixty-five of the adolescents (80%) were female. Table 1 shows the descriptive statistics of the study variables stratified by adolescent gender, as well as the results of between-group comparisons.

The age ranged between 14 and 20 years for adolescents and between 40 and 60 years for their mothers. No significant differences by gender were observed in socio-demographic variables, except for maternal age, with mothers of female adolescents being significantly older than those of male adolescents (*p* = 0.02). Female adolescents reported higher levels of internalizing problems than males (*p* = 0.02), while no significant differences were observed between the two groups in PI variables.

Table 2 shows Spearman’s correlation coefficients among the study variables. Among female adolescents, PI was significantly and positively correlated with both maternal PI and internalizing problems. Among male adolescents, the only significant association was between adolescents’ PI and internalizing problems.

In the multiple linear regression analyses, the association between adolescent’s PI and internalizing problems was statistically significant in females (β = 0.54, *p* ≤ 0.001), but not in males (β = 0.59, *p* = 0.06). The association between mother’s PI and internalizing problems was not statistically significant for either females (β = 0.18, *p* = 0.16) or males (β = −0.41, *p* = 0.19).

Table 3 presents the results of the mediation analyses investigating whether adolescents’ PI mediates the effect of maternal PI on adolescents’ internalizing problems, separately for females and males. When maternal PI was treated as a continuous variable, none of the effects reached statistical significance in either group. For females, model comparisons based on the AIC indicated that models including maternal PI as a dichotomous variable provided a better fit than those including it as a continuous variable. In these models, the total effect of maternal PI on internalizing problems was statistically significant (β = 0.57, *p* = 0.037), and 61% of this effect was mediated by adolescents’ PI (β = 0.36, *p* = 0.045).

In contrast, for males, and based on the AIC, the models with maternal PI as a dichotomous variable did not outperform those with the continuous predictor, and, as in the models with a continuous predictor, no significant effects were observed.

## 4. Discussion

Parental and adolescent PI are known to contribute to various forms of psychopathology, including internalizing problems [20,21,25,28,29,30,31,32,33]. However, it remains unclear whether there is a relationship between parental and adolescent PI, and whether such a relationship may account for the internalizing difficulties observed in adolescents. This study investigated whether adolescent PI serves as a mechanism through which maternal PI influences internalizing symptomatology.

First, the results of this study show that adolescent PI is positively associated with internalizing problems in both females and males. These findings are in line with prior evidence (e.g., [20,22,29,30]) and further support the conceptualization of PI as a transdiagnostic risk factor associated with mental health in adolescence [14,15,16,17,18]. Understanding how adolescent PI develops is therefore essential, as this knowledge may inform more effective intervention and prevention strategies.

Second, our results show that adolescent PI in females is positively and significantly associated with maternal PI, suggesting a potential intergenerational transmission of PI, in particular from mothers to their daughters. To our knowledge, only Moyer and Sandoz [34] have investigated this relationship, reporting no significant association between parental and adolescent PI. As they noted, their null results may be due to their limited sample size and to the use of a parental-specific measure of PI [34]. Indeed, the incremental validity of parenting-specific measures of PI over general measures has yet to be established [47]. In the present study, a general measure of maternal PI (the AAQ-II) was used in a larger sample, and the results supported the hypothesized association between maternal and adolescent PI among females. Further research is warranted to clarify whether the use of parenting-specific PI measures may limit or enhance the utility of these assessment tools [47].

It is worth noting that our findings suggest that the relationship between maternal and adolescent PI may be gender-specific for females. This is of particular interest, as intergenerational transmission of PI in females may help explain, at least partially, the higher prevalence of internalizing disorders typically observed in females (e.g., [4,5,7,9,10,11,12,13]). Interestingly, previous studies have shown that females tend to report higher levels of PI compared to their male peers [37,38], further supporting the need to explore gender as a potential moderator in this pathway.

Nevertheless, caution is warranted when interpreting these gender-related findings. Moyer and Sandoz [34] did not stratify their analyses by gender when examining the relationship between adolescent and maternal PI, which limits the possibility of direct comparisons with this previous research. Furthermore, the lack of significant results among males in the present study may be due to low statistical power, given the small number of male participants. Future studies with larger and more balanced samples are needed to further investigate whether gender-specific pathways exist in the association between adolescent and maternal PI.

Third, the primary aim of this study was to investigate whether adolescent PI mediates the relationship between maternal PI and adolescent internalizing problems. Our results supported this mediating effect for female adolescents. Specifically, adolescent PI partially mediated the effect of maternal PI on internalizing problems. These findings are consistent with the PI model [14,19], which emphasizes that PI is a learned pattern of behavior shaped by various life contexts, including family interactions, social environments, and broader developmental experiences. This may occur, for example, through emotionally avoidant parenting styles, the use of language that promotes experiential avoidance, or behavioral modeling that encourages cognitive fusion [35]. Over time, such processes may shape adolescent’s PI in the face of difficult emotional discomfort which, in turn, may increase psychological distress.

Although dichotomizing maternal PI entails a loss of information and thus represents a methodological limitation, the fact that the model with the dichotomous predictor yielded significant results is theoretically noteworthy and warrants further attention. Theoretically, it is reasonable to assume that the mediation effect of adolescent PI could emerge more clearly when maternal PI is high. Elevated levels of maternal PI may be more likely to foster rigid, avoidant, and emotionally restricted parenting behaviors that could, in turn, be transmitted to adolescents. This possibility suggests that the mediation effect might only become detectable when maternal PI reaches a sufficiently high level to exert a meaningful influence on adolescent outcomes, which could explain why the mediation was observed only when maternal PI was modeled as a dichotomous predictor. Future research could further investigate this possibility by exploring potential non-linear effects of parental PI on adolescent functioning.

Taken together, findings from this study highlight the importance of early interventions targeting PI not only in adolescents, but also in their caregivers. In particular, interventions aimed at reducing maternal PI may indirectly contribute to improving adolescent emotional well-being, especially in girls. Integrating ACT–based approaches [19] within school settings, family-based programs, or parenting interventions could strengthen their preventive impact. Nevertheless, even though ACT has shown to be effective in reducing PI and increasing PF in adult populations (e.g., [17,18]), current evidence on its effectiveness in childhood and adolescence remains limited, and hence further studies are needed to support its applicability in these age groups [31]. In addition, more research is needed to determine whether reductions in parental PI translate into meaningful improvements in adolescent mental health [48].

Several limitations should be taken into account when interpreting the above findings. First, the small sample size—particularly the limited number of male participants—reduced the statistical power of the analyses, potentially limiting the ability to detect significant effects. It also prevented us from controlling for additional socio-demographic variables that may influence the study outcomes. Moreover, the relative homogeneity of our sample, which was mostly drawn from a specific geographical and educational context, limits the extent to which these findings can be generalized to broader populations.

Second, all variables were assessed using self-report questionnaires, which may introduce both methodological (e.g., common method variance) and psychological (e.g., social desirability) biases. This may be particularly relevant for adolescent internalizing problems, for which no parent-report or multi-method assessments were conducted. Furthermore, only mothers participated, as recruitment of fathers proved challenging due to their limited willingness to participate.

Future research could address these limitations by employing multi-method and multi-informant assessment approaches (e.g., [49]), and more diverse and balanced samples in terms of gender and parental roles. In particular, the inclusion of fathers would help clarify whether the observed processes are specific to the mother–daughter dyad or reflect broader parent–child dynamics. Moreover, further investigation could include measures of maternal psychological distress in order to provide a more comprehensive understanding of the interplay between PI and adolescent outcomes [50].

Finally, the cross-sectional design of this study precludes causal interpretations of the observed associations. Future research should also continue to explore the intergenerational dynamics of PI particularly through longitudinal designs that allow for testing causal and developmental pathways over time. Whether adolescent PI mediates the effects of other parental factors (e.g., parenting style, emotion regulation, or stress), and whether contextual factors (e.g., family conflict, social support, and socioeconomic status) moderate these associations, also warrant investigation.

In conclusion, although preliminary and exploratory in nature, this study contributes novel insights into the literature on PI and adolescent mental health. It is the first, to our knowledge, to empirically test the mediating role of adolescent PI in the relationship between maternal PI and internalizing problems, specifically among females. These findings align with the view of PI as a transdiagnostic vulnerability and highlight the importance of targeting both parental and adolescent PI in efforts to prevent and treat internalizing disorders.

## Figures and Tables

**Table 1:** Characteristics of the study sample.

Variable		Female Adolescents N = 65	Male Adolescents N = 16			All N = 81
		**Mean (SD)**	**Mean (SD)**	**t**	*p*-Value *	**Mean (SD)**
Age		16.7 (1.8)	16.5 (2.0)	−0.35	0.73	16.7 (1.8)
Mother’s age		50.6 (4.9)	47.8 (3.8)	−2.45	0.02	50.0 (4.8)
PI		15.7 (6.9)	14.1 (4.9)	−1.08	0.29	15.4 (6.5)
Mother’s PI		18.1 (8.6)	16.7 (6.5)	−0.76	0.46	17.9 (8.2)
Internalizing problems		26.4 (11.5)	18.8 (10.7)	−2.53	0.02	24.9 (11.7)
		**N (%)**	**N (%)**		*p*-value *	**N (%)**
**Adolescent’s nationality**						
	**Italian**	64 (98)	15 (94)		0.358	79 (98)
	**Other**	1 (2)	1 (6)			2 (2)
**Adolescent’s education**						
	**Secondary school**	58 (89)	13 (81)		0.403	71 (88)
	**University**	7 (11)	3 (19)			10 (12)
**Mother’s nationality**						
	**Italian**	61 (94)	15 (94)		1.000	76 (94)
	**Other**	4 (6)	1 (6)			5 (6)
**Mother’s education**						
	**Primary school**	12 (18)	4 (25)		0.433	16 (20)
	**Secondary school**	40 (62)	7 (44)			47 (58)
	**University**	13 (20)	5 (31)			18 (22)
**Mother’s employment**						
	**Employed**	52 (80)	12 (75)		0.734	64 (79)
	**Unemployed**	13 (20)	4 (25)			17 (21)
**Mother’s marital status**						
	**Married/cohabiting**	50 (77)	14 (88)		0.543	64 (79)
	**Divorced/separated**	12 (18)	1 (6)			13 (16)
	**Single**	3 (5)	1 (6)			4 (5)

* Welch’s test for continuous variables and Fischer’s exact test for categorical variables.

**Table 2:** Spearman’s correlation coefficients among the study variables ^+^.

		Mother’s PI	Internalizing Problems
**Female** **adolescents**			
	Internalizing problems	0.24	-
	PI	0.31 *	0.54 **
**Male** **adolescents**			
	Internalizing problems	−0.38	-
	PI	−0.38	0.55 *

^+^ PI: Psychological inflexibility. * *p* ≤ 0.05, ** *p* ≤ 0.001.

**Table 3:** Analysis of the role of adolescent’s PI as mediator of the effect of mother’s PI (as continuous or dichotomous predictor) on internalizing problems ^+^.

	Mother’s PI Continuous	Mother’s PI Dichotomous
**Females**	*β* (95% CI)	*β* (95% CI)
Direct effect	0.05 (−0.18, 0.27)	0.21 (−0.28, 0.72)
Mediated effect	0.13 (−0.02, 0.30)	0.36 (0.07, 0.73) *
Total effect	0.18 (−0.10, 0.43)	0.57 (0.04, 1.06) *
Proportion of mediated effect	0.63 (−2.79, 4.58)	0.61 (0.03, 2.42) *
**Males**		
Direct effect	−0.23 (−0.83, 0.41)	−0.44 (−1.43, 0.61)
Mediated effect	−0.19 (−0.72, 0.13)	−0.24 (−1.03, 0.31)
Total effect	−0.42 (−1.02, 0.23)	−0.68 (−1.64, 0.38)
Proportion of mediated effect	0.34 (−2.34, 3.61)	0.26 (−1.80, 3.53)

^+^ All variables were standardized. The models were adjusted for mother’s and adolescent’ age. *β*: coefficient, CI: Confidence Interval. * *p* ≤ 0.05.

## Data Availability

The data presented in this study are available on request from the corresponding author. The data are not publicly available due to privacy.

## Abbreviations

The following abbreviations are used in this manuscript:

PI	Psychological Inflexibility
ACT	Acceptance and Commitment Therapy
YSR	Youth Self Report
AFQ-Y	Avoidance and Fusion Questionnaire for Youth
AAQ-II	Acceptance and Action Questionnaire-II
SD	Standard Deviation
CI	Confidence Interval
*β*	standardized coefficient
